# Field evaluation of a locally produced rapid diagnostic test for early detection of cholera in Bangladesh

**DOI:** 10.1371/journal.pntd.0007124

**Published:** 2019-01-31

**Authors:** Md. Taufiqul Islam, Ashraful Islam Khan, Md. Abu Sayeed, Jakia Amin, Kamrul Islam, Nur Alam, Nishat Sultana, Noor Jahan, Md. Mahbubur Rashid, Zahid Hasan Khan, Mazharul Islam Zion, Mokibul Hassan Afrad, Shah Alam Siddique, Farhana Khanam, Yasmin Ara Begum, Muhammad Shariful Islam, Firdausi Qadri

**Affiliations:** 1 International Centre for Diarrheal Disease Research, Bangladesh (icddr,b), Dhaka, Bangladesh; 2 Incepta Pharmaceuticals Ltd., Dhaka, Bangladesh; Yale University Yale School of Public Health, UNITED STATES

## Abstract

**Background:**

Cholera remains a substantial health burden in Asia and Africa particularly in resource poor settings. The standard procedures to identify the etiological organism *V*. *cholerae* are isolation from microbiological culture from stool as well as Polymerase Chain Reaction (PCR). Both the processes are highly lab oriented, labor extensive, time consuming, and expensive. In an effort to control for outbreaks and epidemics; an effective, convenient, quick and relatively less expensive detection method is imperative, without compromising the sensitivity and specificity that exists at present. The objective of this component of the study was to evaluate the effectiveness of a locally produced rapid diagnostic test (RDT) for cholera diagnosis.

**Methods:**

In Bangladesh, nationwide cholera surveillance is ongoing in 22 hospitals covering all 8 divisions of the country since June, 2016. In the surveillance, stool samples have been collected from patients presenting to hospitals with acute watery diarrhea. Crystal VC^TM^ (Span diagnostics, India) and Cholkit (locally produced RDT) have been used to detect *V*. *cholerae* from stool samples. Samples have also been sent to the main laboratory at icddr,b where the culture based isolation is routinely performed. All the tests were carried out for both direct and enriched stool samples. RDT sensitivity and specificity were calculated using stool culture as the gold standard.

**Results:**

A total of 7720 samples were tested. Among these, 5865 samples were solely tested with Crystal VC and 1355 samples with Cholkit whereas 381 samples were tested with both the RDTs. In comparison with culture, direct testing with Crystal VC showed a sensitivity of 72% (95% CI: 50.6% to 87.9%) and specificity of 86.8% (95% CI: 82.8% to 90.1%). After enrichment the sensitivity and specificity was 68% (95% CI: 46.5% to 85.1%) and 97.5% (95% CI: 95.3% to 98.8%) respectively. The direct Cholkit test showed sensitivity of 76% (95% CI: 54.9% to 90.6%) and specificity of 90.2% (95% CI: 86.6% to 93.1%).

**Conclusion:**

This evaluation has demonstrated that the sensitivity and specificity of Cholkit is similar to the commercially available test, Crystal VC when used in field settings for detecting *V*. *cholerae* from stool specimens. The findings from this study suggest that the Cholkit could be a possible alternative for cholera endemic regions where *V*. *cholerae* O1 is the major causative organism causing cholera.

## Author summary

Cholera still remains a burning public health issue in the developing world. Microbiological culture is the gold standard method for cholera diagnosis. However, it requires well equipped laboratories and 24–72 hours’ time for the isolation of pathogens, which may not be feasible for hard to reach areas and during epidemics or seasonal outbreaks. For the effective control of disease outbreaks, detection methods should be both quick and easy without sacrificing specificity and sensitivity. Rapid diagnostic test for cholera could be a potential alternative for early detection of the disease. Addressing this issue in our study, we report the performance of a rapid diagnostic test (RDT), Cholkit for the diagnosis of cholera cases using stool obtained in field settings and the assessment of its performance with those of microbial culture and Crystal VC assay, a commercially available dipstick.

## Introduction

Even with the development of modern established treatments and preventative measures, cholera still remains a major health burden in low-income countries with limited resources, particularly in the developing world. Cholera is a water-borne infectious disease which can be characterized by life-threatening secretory diarrhea, often accompanied by numerous voluminous watery stools and vomiting [1]. Clinical consequences range from asymptomatic to severe disease with massive watery diarrhea which may become fatal if untreated [2]. Globally, an estimated 1.3 billion people are at risk of cholera where India and Bangladesh jointly constitute the largest share of population at risk. In Bangladesh, according to estimations, at least 66 million people are at risk of cholera with an estimated 109,052 cholera cases annually [3]. While many infections can result in only mild symptoms, at least 300,000 severe cases occur annually which are severe enough requiring hospitalisation [4]. The causative agent of cholera at present is *Vibrio cholerae* O1, a Gram-negative pathogen. To date, more than 200 *V*. *cholerae* serogroups have been identified where most serogroups are non-pathogenic. Only isolates of serogroup O1 (consisting of two biotypes known as ‘classical’ and ‘El Tor’ and the serotypes Ogawa and Inaba) and O139 have been reported to be pathogenic and cause cholera epidemics and pandemics. However, in the last decade no epidemics due to *V*. *cholerae* O139 have been reported and only sporadic clinical cases have been observed [5].

Stool culture remains the reference method for laboratory surveillance of cholera though the sensitivity of direct stool culture is not 100% and depends on the concentration of *V*. *cholerae* (10^6^–10^7^ CFU) in stool specimens [6–10]. Moreover, due to limited facilities in peripheral and field sites, diagnosis is a major hindrance for early detection of cholera in endemic regions or during a cholera epidemic. The routine culture also costs approximately 6–8 USD/case [8] and the procedure involves isolation of the bacteria, routine microbiological and biochemical analyses which is lengthy and requires about 24–72 hours. Additionally, microbiological facilities are often not feasible in remote locations and transport to the closest sufficiently equipped laboratory may add further costs. Various molecular-based techniques have been developed including PCR for the rapid detection of virulence and regulatory genes [11]. Although the specificity of PCR method is relatively high, it requires expensive equipment and technical expertise which may is very often not available in small laboratories or field settings.

For the effective control of disease outbreaks, diagnostic methods should be both quick and easy without sacrificing specificity and sensitivity of detection. RDT for cholera could be a potential alternative with advantages such as it is rapid, requires minimum training, easy to use and interpret, can be stored at ambient temperature, reasonably priced and can be deployed widely for early confirmation of cholera outbreaks. One of the most recent cholera RDTs currently available in the market is Crystal VC™ (Span Diagnostics Ltd, Surat, India), a dipstick assay initially developed by the Institut Pasteur which is now being produced by Span Diagnostics (Surat, Guzarat, India). The assay relies on the detection of the lipopolysaccharide (LPS) antigen of both *V*. *cholerae* O1 and O139 serogroups by monoclonal antibodies based on a one-step vertical-flow immunochromatography principle. Crystal VC has shown sensitivity ranging from 94–100%, and a specificity range of 84–100% [9, 12–14]. However, the O1 and O139 together in Crystal VC lead to lower specificity. Recently, another RDT named ‘Cholkit’ has been developed by our group. Cholkit is a lateral flow immunochromatography test for the qualitative determination of LPS antigen of only *Vibrio cholerae* O1 serogroup using monoclonal antibody specific to *V*. *cholerae* O1 [15]. The objective of this study was to evaluate the performance of the RDT Cholkit and compare the performance with Crystal VC assay, a commercially available RDT designed to detect *V*. *cholerae* O1 and O139.

## Methods

### Ethical statement

The study protocol was approved by the Research Review Committee (RRC) and Ethical Review Committee (ERC) at the icddr,b. Informed written consent was taken from adult patients, or guardians on behalf of children.

### Study sites and population

**Fig 1:**
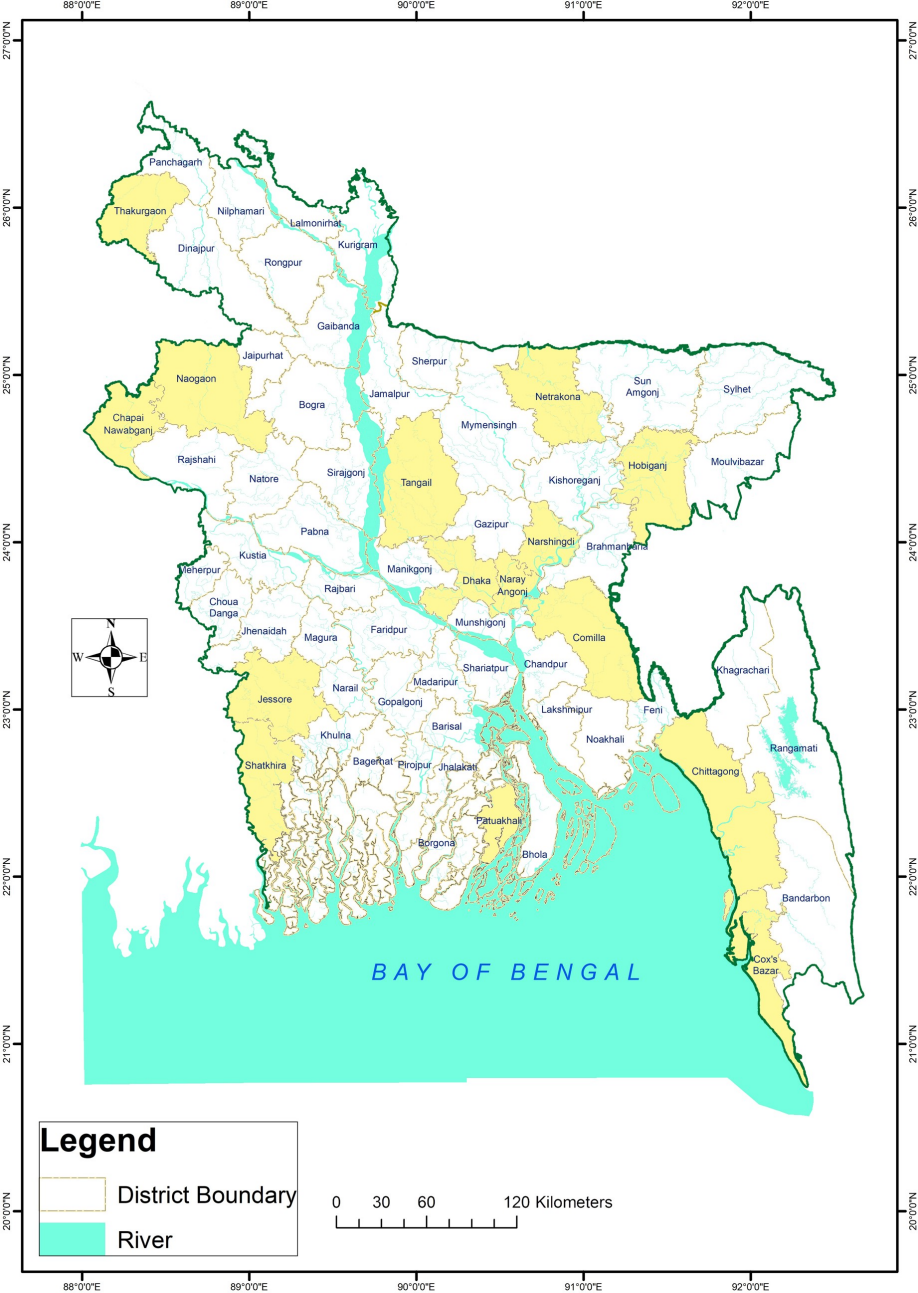
Surveillance sites. Map has been created by Arc GIS v 10.6.

Since 2016, icddr,b has been running a nationwide enteric disease surveillance in collaboration with Institute of Epidemiology Disease Control & Research (IEDCR) under the Government of Bangladesh (GoB). The surveillance is being conducted in different districts comprising of 22 sentinel sites (health facilities), covering all 8 divisions across Bangladesh (Fig 1). Stool samples were obtained from individuals seeking treatment with complaints of acute watery diarrhea. A diarrheal visit was defined as a patient (age > 2 months) attending hospital with 3 or more loose or liquid stools in last 24 hours or less than 3 loose/ liquid stools causing dehydration; or at least one bloody loose stool in last 24 hours, as well as (age < 2 months) changed stool habit from usual pattern in terms of frequency (more than usual number of purging) or nature of stool (more water than fecal matter).

Patients presented with acute watery diarrhea were requested to provide a stool sample. Freshly collected stool samples were immediately used for the direct dipstick assay at the sentinel sites. Fecal specimens were concurrently enriched overnight at 37°C in alkaline peptone water (APW) (1% peptone, 1% NaCl; pH-8.5) and dipstick assays were performed on the following day. For culture, stool samples were placed into the Cary Blair transport medium and transported to the icddr,b laboratory fortnightly by maintaining the cold chain (2−8^0^ C). Initially all stool specimens (n = 381) were tested with both Crystal VC and Cholkit simultaneously. After observing similar performance of two RDTs, the kits were separately provided in different field sites.

### Stool culture

Conventional stool culture was carried out by streaking stool directly on selective TTGA (taurocholate-tellurite gelatin agar) plates, and plates were incubated overnight at 37°C. Enrichment was performed in APW overnight at 37°C, followed by plating on TTGA to isolate *V*. *cholerae*. Colonies morphologically consistent with *V*. *cholerae* were tested for agglutination reaction with monoclonal antibodies specific to *V*. *cholerae* serovar O1 (Ogawa or Inaba) and O139.

### Crystal VC dipstick test

#### Direct stool

Five drops (200–250 μl) of liquid stool were added into the sample processing vial and mixed gently. Four drops of the processed sample were then put in a test tube. The Crystal VC test strip was dipped into the tube and the results were interpreted after 15 min according to the manufacturer’s protocol.

#### Enriched stool

For enrichment, twelve drops of liquid stool were added into enrichment vial containing APW and kept overnight at ambient temperature. Five drops of enriched stool were then added to the sample processing vial. The testing was performed according to the manufacturer instructions.

### Cholkit dipstick test

#### Direct stool

Cholkit test was performed as previously describe by Sayeed et al [15]. Briefly, five drops of watery stool were transferred into sample processing vial and the Cholkit strip was dipped into it for 15 min; the test line and/or control line appeared as a red color. Appearance of both lines indicated that the sample was positive for *V*. *cholerae* O1; appearance of only the control line but not the test line indicated a negative result for the test.

#### Enriched stool

For enrichment, twelve drops of liquid stool were added into enrichment vial containing APW and kept overnight at ambient temperature. Five drops of enriched stool were transferred into sample processing vial and tested the strip.

### Data analysis

Clinical and sociodemographic data were collected as per the original protocol requirement. Data were checked and then entered into the visual studio version 10.0 (Texas, USA). After completing data entry, data were transferred into the SQL server 2008. Data consistency was checked using SQL query. The primary endpoint was the assessment of the performance of the RDT using microbiological stool culture result as the gold standard for comparison. Sensitivity (true-positive or TP rate) was defined as the probability that patients with laboratory-confirmed cholera had a positive RDT. Specificity (true-negative or TN rate) was defined as the probability that patients with no laboratory-confirmed cholera had a negative RDT. The positive predictive value (PPV) was the probability that patients with a positive RDT had *V*. *cholerae* isolated from stool culture. The negative predictive value (NPV) was the probability that patients with a negative RDT had no *V*. *cholerae* isolated from a stool culture. Proportion test statistics was used for calculating p-values to distinguish the difference between two RDT kits in terms of sensitivity and specificity.

Statistical analyses were conducted using STATA version 13 (USA). Sensitivity and specificity were determined based on the comparison of Cholkit and Crystal VC results with the lab culture test and presented as percentages. Along with the percentages of sensitivity and specificity, 95% Clopper-Pearson confidence intervals (CIs) were as estimated for better predictions.

## Results

**Fig 2:**
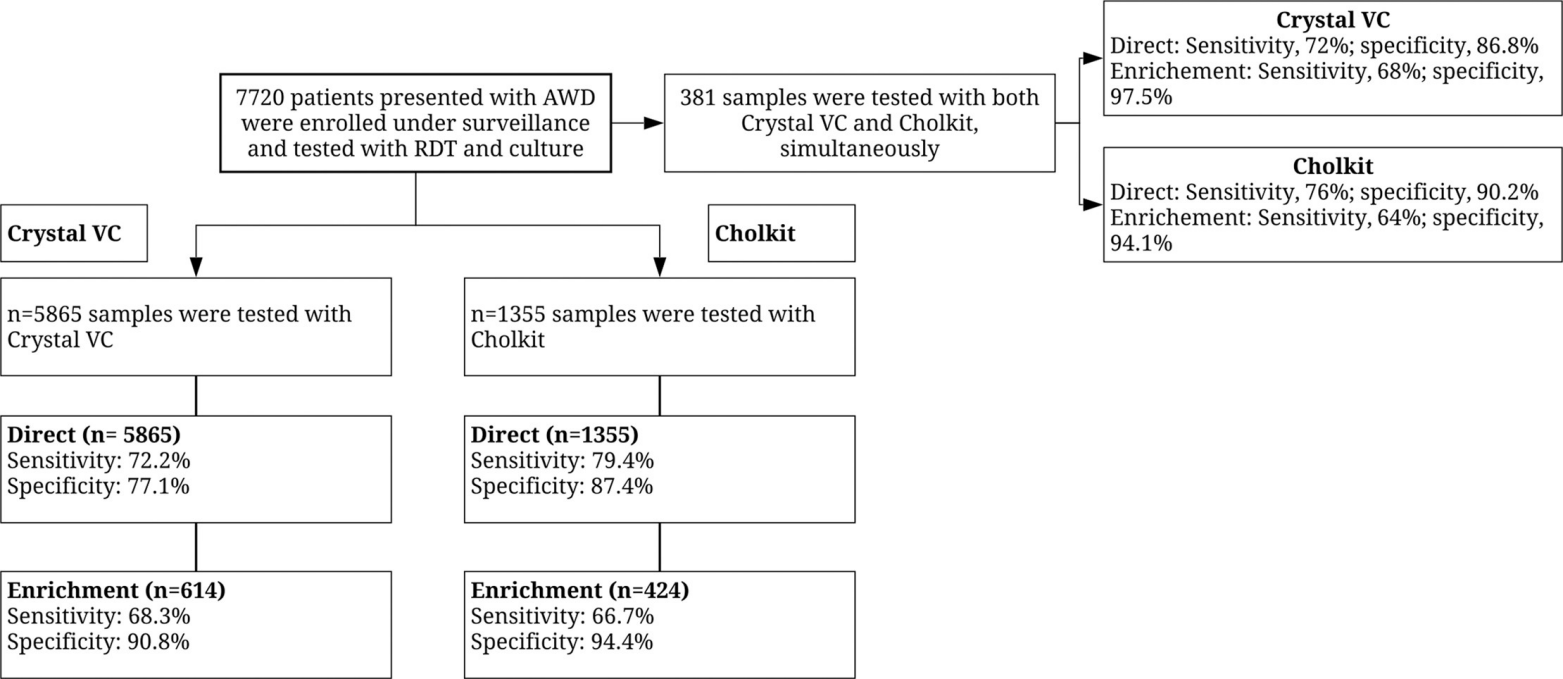
Flow of study participants.

From 22 sentinel surveillance sites, a total of 7220 patients who presented with acute watery diarrhea were recruited into the study and analyzed to evaluate the performance of two RDT Kits (Fig 2). Among them 50% were from <5 years age group and 5% from 5–17 years old and the rest 45% from those who were older. Mean age of the participants was 18.75 years, and 55% were male. Among them, 381 stool samples (both direct and enriched stool) were tested by using both Cholkit and Crystal VC at the field sites, and the performance was compared with microbiological culture.

Amongst 381 stools, *V*. *cholerae* was isolated from 25 (6.6%) samples by culture. Positivity by Crystal VC with direct and enriched sample was 65/381 (17.1%) and 26/381 (6.8%), respectively, whereas Cholkit with direct and enriched sample was positive for 54/381 (14.2%) and 37/381 (9.7%) respectively (Table 1). Crystal VC on direct stool showed a sensitivity of 72.0% (95% CI: 50.6% to 87.9%), specificity of 86.8% (95% CI: 82.8% to 90.1%) and after enrichment the sensitivity and specificity were 68% (95% CI: 46.5% to 85.1%) and 97.5% (95% CI: 95.3% to 98.8%) respectively. Negative predictive values (NPV) of Crystal VC were similar; however, we found different positive predictive values (PPV) 27.7% and 65.4% on direct and enriched stool respectively. Test results on direct sample of Cholkit revealed a sensitivity of 76.0% (95% CI: 54.9% to 90.6%) and specificity 90.2% (95% CI: 86.6% to 93.1%) while enrichment revealed 64% (95% CI: 42.5% to 82.0%) and 94.1% (95% CI: 91.1% to 96.3%) respectively. The sensitivity and specificity of the RDTs using either direct or enrichment methods, were not found to be different (p>0.05). The PPVs of Cholkit was 35.2% and 43.2% on fresh and enriched samples, whereas NPVs were similar (Table 2).

**Table 1:** Results on direct and enriched sample through RDTs in field settings where both RDTs were used simultaneously.

RDT results	Culture results		
	Positive	Negative	Total
Crystal VC Direct			
Positive	18	47	65 (17.1%)
Negative	7	309	316
Total	25	356	381
Cholkit Direct			
Positive	19	35	54 (14.2%)
Negative	6	321	327
Total	25	356	381
Crystal VC Enrichment			
Positive	17	9	26 (6.8%)
Negative	8	347	355
Total	25	356	381
Cholkit Enrichment			
Positive	16	21	37 (9.7%)
Negative	9	335	344
Total	25	356	381

**Table 2:** Diagnostic performance of direct and enriched RDTs in field settings where both RDTs were used simultaneously.

RDTs	Sensitivity % (95% CI)	p-value	Specificity % (95% CI)	p-value	PPV % (95% CI)	NPV % (95% CI)
Crystal VC Direct	72.0 (50.6, 87.9)	0.747	86.8 (82.8, 90.1)	0.159	27.7 (21.1, 35.5)	97.8 (95.9, 98.8)
Cholkit Direct	76.0 (54.9, 90.6)		90.2 (86.6, 93.1)		35.2 (27.0, 44.4)	98.2 (96.4, 99.1)
Crystal VC Enrichment	68.0 (46.5, 85.1)	0.765	97.5 (95.3, 98.8)	0.025	65.4 (48.4, 79.2)	97.8 (96.1, 98.7)
Cholkit Enrichment	64.0 (42.5, 82.0)		94.1 (91.1, 96.3)		43.2 (31.4, 55.9)	97.4 (95.7, 98.4)

A total of 5,865 direct stool samples and a subset of 614 enriched stools were tested with Crystal VC. On the other hand, 1,355 direct stools and a subset of 424 enriched samples were tested with Cholkit (Table 3). The sensitivity and specificity of Cholkit with direct stool was 79.4% (95% CI: 62.1% to 91.3%), and 87.4% (95% CI: 85.5% to 89.1%) respectively, while the sensitivity and specificity of Cholkit with enriched stool was 66.7% (95% CI: 47.2% to 82.7%), and 94.4% (95% CI: 91.7% to 96.5%), respectively. PPVs were 13.9% on direct stool and 47.6% on enriched sample, whereas NPVs showed similar result on both. In contrast, sensitivity and specificity of Crystal VC with direct stool was 72.2% (95% CI: 64.6% to 78.9%) and 77.1% (95% CI: 75.9% to 78.2%) respectively, while the sensitivity and specificity of Crystal VC was respectively 68.3% (95% CI: 51.9% to 81.9%) and 90.8% (95% CI: 88.1% to 92.9%) with enriched stool. The results of NPVs are almost similar, while PPVs are 8.2% on fresh stool and 34.6% on enriched sample (Table 4).

**Table 3:** Test results on direct and enriched sample using RDTs in field settings where both RDTs were used independently.

RDT Results	Culture results						
	Direct			Enrichment		
	Positive	Negative	Total	Positive	Negative	Total
Crystal VC						
Positive	117	1,308	1,425	28	53	81
Negative	45	4,395	4,440	13	520	533
Total	162	5,703	5,865	41	573	614
Cholkit						
Positive	27	166	193	20	22	42
Negative	7	1,155	1,162	10	372	382
Total	34	1,321	1,355	30	394	424

**Table 4:** Diagnostic performance of direct and enriched RDTs in field settings where RDTs were used independently.

RDTs	Sensitivity % (95% CI)	Specificity % (95% CI)	PPV % (95% CI)	NPV % (95% CI)
Crystal VC Direct	72.2 (64.6, 78.9)	77.1 (75.9,78.2)	8.2 (7.4, 9.1)	98.9 (98.7, 99.2)
Crystal VC Enrichment	68.3 (51.9, 81.9)	90.8 (88.1, 92.9)	34.6 (27.5, 42.4)	97.6 (96.2, 98.4)
Cholkit Direct	79.4 (62.1, 91.3)	87.4 (85.5, 89.1)	13.9 (11.5, 16.9)	99.4 (98.8, 99.7)
Cholkit Enrichment	66.7 (47.2, 82.7)	94.4 (91.7, 96.5)	47.6 (36.0, 59.5)	97.4(95.7, 98.4)

## Discussion

Rapid and accurate diagnosis of cholera at the earliest stages of an epidemic is a key feature to assist in early management of cholera outbreaks. Thus, there is a pressing need for simple and inexpensive RDT to correctly identify patients with cholera. Till date many RDTs for early detection of cholera have been evaluated [16]. Although, the sensitivity and specificity of these tests were substantial, all may not be suitable for use in the field settings. Crystal VC has been the most widely used as cholera RDT till date. Although Crystal VC is well regarded for higher sensitivity, the presence of O139 in the kit has been reported to lead to lower specificity. Recently our group has developed Cholkit RDT which has showed similar sensitivity but improved specificity compared to Crystal VC in the laboratory settings (Sayeed 2018). This study was conducted to evaluate Cholkit in field settings and compare its performance with Crystal VC.

We tested both RDTs with diarrheal stools obtained from our ongoing cholera surveillance studies. Initially, we tested both RDTs simultaneously in the field sites. Analysis with 381 diarrheal stools showed similar sensitivity and specificity. Thereafter, the kits were distributed separately in the field sites. In the field settings, Cholkit showed similar sensitivity and specificity as Crystal VC. Crystal VC detect both *V*. *cholerae* O1 and *V*. *cholerae* O139. In contrast, the newly developed RDT, Cholkit only detects *V*. *cholerae* O1. *V*. *cholerae* O1 is responsible for the majority of cholera outbreaks worldwide while *V*. *cholerae* O139 is confined to Southeast Asia and has not been involved or reported in outbreaks for more than a decade [5]. The O139 serogroup was recognized first in Bangladesh in 1992 and in nearby Southeast Asian countries [17, 18]. Since then there was only one reported outbreak with O139 serogroup in Dhaka, Bangladesh in 2002 [19]. Since then the O139 serogroup has appeared sporadically in clinical and environmental samples in Bangladesh. However, no small or large scale outbreak has been reported due to O139 [5, 20]. During epidemics where *V*. *cholerae* O1 is the responsible strain, in endemic areas, or in surveillance studies where *V*. *cholerae* O1 is the only prevalent strain; testing cholera stool with Crystal VC may create misleading interpretation and ambiguity in calculating the specificity due to the false positive result of *V*. *cholerae* O139 and consequently decrease its specificity. In accordance with our observation, evaluation of Crystal VC in field settings conducted by Ley B et al also reported similar false positive *V*. *cholerae* O139 [21] where the authors speculated that field workers may often over-interpret faint test lines as positive for *V*. *cholerae* O139. Moreover, we also cannot exclude the possibility of false positive O139 results from stool if *V*. *cholerae* O1 concentration is high in stool. Considering that, Cholkit might overcome this limitation because it does not have a test band for *V*. *cholerae* O139 and can be a suitable RDT as an alternative to Crystal VC during the predominant *V*. *cholerae* O1 era. However local production of RDT will also reduce the cost and its accessibility in Bangladesh.

This study has a number of limitations. First, the study did not assess whether the RDT results were affected by the level of skill of the technician and previous intake of antibiotics or intravenous fluids. Compared to the laboratory validation of Cholkit conducted by Sayeed et al [15], we observed lower sensitivity and specificity for both RDTs in the field settings. Previously, Kalluri et al have assessed the impact of the technician’s qualification on the performance of Crystal VC [22]. The reported RDT sensitivities of 94% and 93% when carried out by laboratory technicians and field workers respectively, were similar, but RDT specificity was higher when performed by the technicians (76% versus 67%) [13]. Harris et al and Mukherjee et al have also reported similar observations [8, 23]. Second, while confirmation of *V*. *cholerae* isolates was performed at the icddr,b laboratory, culture-negative stool samples were not validated further. In particular, we did not perform PCR testing on our RDT-positive, culture-negative samples. Bhuiyan et al [12] reported five stool samples by multiplex PCR that were O1 dipstick positive but culture-negative and found that all five were negative by PCR, indicating that the five dipstick-positive results were false positives. Thus, we cannot entirely exclude the possibility of false negativity by stool culture. Third, Alam et al pointed out that the dipstick may detect non-culturable forms of *V*. *cholerae* that have transformed into a coccoid form due to unfavorable intra-host conditions, such as antibiotic treatment prior to testing [24]. Lastly, we were not able to perform the cost analysis since Cholkit is not yet available commercially. However, according to the manufacturer, the cost of the locally produced RDT may be less compared to the commercially available RDT Crystal VC, since only it is based on a single monoclonal antibody.

Early diagnosis of cholera in an outbreak and endemic settings is of substantial public health importance. Moreover, rapid and correct detection of cholera cases in the initial stages of an outbreak is critical for containment of the infection. The RDT Cholkit has shown comparable performance to Crystal VC in the field settings. In resource -limited settings where culture facility is not readily available, Cholkit has good utility and can potentially be used as an early warning tool for cholera outbreaks in the field.

### Conclusion

Our study demonstrated that the RDT Cholkit, locally developed in Bangladesh is comparable to Crystal VC in terms of sensitivity and specificity and can be used for monitoring cholera hotspots and epidemics. The kit will also be relatively cheaper than the commercially available RDT in the market. The Cholkit has only monoclonal antibody that detects *V*. *cholerae* O1. In a cholera endemic region like Bangladesh where *V*. *cholerae* O1 is the only prevalent strain, it is more efficient to have a test available for the O1 serogroup only. The study demonstrates the feasibility of using RDTs for monitoring cholera in resource poor as well as in hard to reach areas. This analysis also demonstrates the presence of cholera hotspots in different parts of Bangladesh in the surveillance carried out in 8 divisions of the country. However, confirmation of the RDT tests with bacteriological culture was also carried out to further strengthen and confirm the results. The information obtained from this study will be useful for planning preventive measures for eliminating cholera in Bangladesh which is an agenda for the global road map of ending cholera by 2030. In conclusion, our data shows that cholera RDTs will be helpful in predicting the population based incidence of cholera in the country and this information can also be utilized in other countries endemic or having epidemic potentials.

## Supporting information

S1 Table Data on sensitivity and specificity of RDTs in all surveillance sites. (DOCX)

S1 Appendix STROBE statement—Checklist of items. (DOCX)
