# Supplementary material for: Field evaluation of a locally produced rapid diagnostic test for early detection of cholera in Bangladesh
Source: PLoS Negl Trop Dis. 2019 Jan 31;13(1):e0007124. doi: 10.1371/journal.pntd.0007124 (PMC6372204; doi:10.1371/journal.pntd.0007124)
Supplement: S1 Table — (DOCX) [file pntd.0007124.s001.docx]

S1 Table: Data on sensitivity and specificity of RDTs in all surveillance sites

| Surveillance Sites | Crystal VC Direct | | | Crystal VC Enrichment | | | Cholkit Direct | | | Cholkit Enrichment | | |
| --- | --- | --- | --- | --- | --- | --- | --- | --- | --- | --- | --- | --- |
|  | n | Sensitivity % (95% CI) | Specificity % (95% CI) | n | Sensitivity % (95% CI) | Specificity % (95% CI) | n | Sensitivity % (95% CI) | Specificity % (95% CI) | n | Sensitivity % (95% CI) | Specificity % (95% CI) |
| Narshindi | 213 | - | - | 4 | - | - | 9 | - | - | 4 | - | - |
| Habiganj | 588 | 55 (36, 71) | 54 (50, 58) | 22 | - | - | 25 | - | 96 (79, 99) | 10 | - | - |
| Cox's Bazar | 579 | 62 (50, 73) | 63 (58, 67) | 84 | 75 (19, 99) | 71 (60, 81) | 22 | 100 (3, 100) | 62 (38, 82) | 12 | 100 (3, 100) | 73 (39, 93) |
| Naogaon | 475 | 32 (15, 54) | 77 (73, 81) | 14 | - | - | 27 | 50 (2, 98) | 88 (69, 97) | 10 | - | - |
| Patuakhali | 297 | 75 (35, 97) | 80 (75, 85) | 18 | 100 (3, 100) | 100 (80, 100) | 19 | 100 (3, 100) | 94 (73, 99) | 10 | 100 (3, 100) | 100 (66, 100) |
| Thakurgaon | 320 | 14 (1, 58) | 83 (78, 87) | 13 | - | - | 15 | - | - | 7 | - | - |
| Shatkhira | 338 | - | - | 11 | - | - | 9 | - | - | 7 | - | - |
| DMCH, Dhaka | 20 | - | - | 13 | - | - | 165 | 100 (3, 100) | 98 (94, 99) | 9 | - | - |
| UAMC&H, Dhaka | 15 | - | - | 9 | - | - | 189 | 57 (18, 90) | 90 (85, 94) | 7 | - | - |
| BITID, Chittagang | 345 | 54 (40, 67) | 63 (57, 69) | 50 | 67 (38, 88) | 77 (60, 90) | 14 | 71 (29, 96) | 71 (29, 96) | 7 | 60 (15, 94) | 100 (16, 100) |
| Tangail | 713 | 37 (21, 55) | 82 (79, 85) | 154 | - | 96 (92, 98) | 143 | - | 84 (77, 89) | 110 | - | 93 (86, 97) |
| Narayanganj | 171 | 71 (49, 87) | 80 (73, 86) | 83 | 78 (52, 94) | 88 (77, 95) | 470 | 82 (68, 92) | 83 (79, 86) | 116 | 75 (51, 91) | 90 (82, 95) |
| Chuadanga | 440 | 45 (27, 64) | 79 (75, 83) | 47 | - | 98 (88, 99) | 62 | - | 98 (90, 99) | 47 | - | 98 (88, 99) |
| Meherpur | 523 | 67 (38, 88) | 89 (86, 92) | 71 | - | - | 102 | 100 (3, 100) | 98 (93, 99) | 68 | - | - |
| Comilla | 260 | 60 (45, 74) | 81 (75, 86) | 21 | - | - | 0 | - | - | 0 | - | - |
| Chowgacha, Jessore | 0 | - | - | 0 | - | - | 84 | 100 (3, 100) | 97 (89, 99) | 0 | - | - |
| Kushtia | 398 | 42 (23, 63) | 85 (81, 89) | 0 | - | - | 0 | - | - | 0 | - | - |
| Madan | 86 | - | - | 0 | - | - | 0 | - | - | 0 | - | - |
| Chhatak | 45 | - | - | 0 | - | - | 0 | - | - | 0 | - | - |
| Mathbariya | 30 | 100 (16, 100) | 93 (4, 53) | 0 | - | - | 0 | - | - | 0 | - | - |
| Bakerganj | 9 | - | - | 0 | - | - | 0 | - | - | 0 | - | - |
| Shibganj | 0 | - | - | 0 | - | - | 0 | - | - | 0 | - | - |
| Total | 5865 |  |  | 614 |  |  | 1355 |  |  | 424 |  |  |
